# Be Aggressive! Amorphous Excipients Enabling Single-Step Freeze-Drying of Monoclonal Antibody Formulations

**DOI:** 10.3390/pharmaceutics11110616

**Published:** 2019-11-17

**Authors:** Christina Haeuser, Pierre Goldbach, Joerg Huwyler, Wolfgang Friess, Andrea Allmendinger

**Affiliations:** 1 Late Stage Pharmaceutical and Processing Development, Pharmaceutical Development & Supplies, Pharma Technical Development Biologics EU, F. Hoffmann-La Roche Ltd., 4070 Basel, Switzerland; christina.haeuser@roche.com (C.H.); pierre.goldbach@roche.com (P.G.); 2 Division of Pharmaceutical Technology, Department of Pharmaceutical Sciences, University of Basel, 4056 Basel, Switzerland; joerg.huwyler@unibas.ch; 3 Pharmaceutical Technology and Biopharmaceutics, Department of Pharmacy, Ludwig-Maximilians-University Munich, 81377 Munich, Germany; Wolfgang.Friess@lrz.uni-muenchen.de

**Keywords:** glass transition, collapse, freeze-drying, cyclodextrin, antibody, cycle optimization, single-step freeze-drying

## Abstract

Short freeze-drying cycles for biopharmaceuticals are desirable. Formulations containing an amorphous disaccharide, such as sucrose, are prone to collapse upon aggressive primary drying at higher shelf temperature. We used 2-hydroxypropyl-betacyclodextrin (HPBCD) in combination with sucrose and polyvinylpyrrolidone (PVP) to develop an aggressive lyophilization cycle for low concentration monoclonal antibody (mAb) formulations. Glass transition temperature and collapse temperature of the formulations were determined, and increasingly aggressive cycle parameters were applied. Using a shelf temperature of +30 °C during primary drying, the concept of combining sublimation and desorption of water in a single drying step was investigated. Cake appearance was evaluated visually and by micro-computed tomography. Lyophilisates were further analyzed for reconstitution time, specific surface area, residual moisture, and glass transition temperature. We demonstrated the applicability of single-step freeze-drying, shortening the total cycle time by 50% and providing elegant lyophilisates for pure HPBCD and HPBCD/sucrose formulations. HPBCD/PVP/sucrose showed minor dents, while good mAb stability at 10 mg/mL was obtained for HPBCD/sucrose and HPBCD/PVP/sucrose when stored at 40 °C for 3 months. We conclude that HPBCD-based formulations in combination with sucrose are highly attractive, enabling aggressive, single-step freeze-drying of low concentration mAb formulations, while maintaining elegant lyophilisates and ensuring protein stability at the same time.

## 1. Introduction

Freeze-drying is frequently used to manufacture drug products of proteins which are unstable as liquids. Proteins are generally more stable in the dried immobilized state as physical (e.g., aggregation) and chemical (e.g., hydrolysis) degradation mechanisms are slowed down. Although around 40% of all biopharmaceuticals are freeze-dried, liquid formulations are often preferred due to a significantly less complex manufacturing process [1,2]. Freeze-drying is a time-consuming low throughput batch process which usually takes several days up to weeks [3], requires much energy, and is ultimately costly. Freeze-drying consists of three process steps, (i) freezing, (ii) primary drying, where crystallized water is removed under vacuum by sublimation, and (iii) secondary drying, where desorption of the bound water takes place. Primary drying is the most time consuming of the three steps. Hence, efforts to optimize the lyophilization cycle time often focus on the primary drying step.

During primary drying it is important that the product temperature (*T*_p_) stays below the critical formulation temperature to avoid collapse. Collapse may [4,5] or may not [6,7] be detrimental to the storage stability of monoclonal antibodies, but in any case is often considered as a defect of the drug product, which might lead to rejects during 100% visual inspection [8]. The collapse temperature (*T*_c_) is typically 1–3 °C above the glass transition temperature of the maximally freeze concentrated solution (*T*_g_’) of an amorphous formulation. Disaccharides such as sucrose and trehalose, which are commonly used in freeze-dried antibody formulations [1], have low *T*_g_’ and *T*_c_ values of around −28 to −32 °C [9]. This makes low pressure and shelf temperature (*T*_s_) during primary drying necessary, resulting in long primary drying time.

Efforts to shorten freeze-drying cycle time have re-gained attraction and different approaches are described in recent literature. For example, microwave assisted freeze-drying enables much faster primary drying compared to conventional freeze-drying [10]. With regards to conventional freeze-drying, it is well-known that increasing *T*_p_ significantly impacts cycle time, e.g., an increase of *T*_p_ by 1 °C may shorten primary drying by 10% [3]. In this light, Colandene et al., Bjelosevic et al., and Depaz et al. successfully optimized primary drying by freeze-drying above *T*_g_’ without introducing collapse [11,12,13]. However, this approach is only applicable for formulations with high protein concentrations or high protein to sugar ratios, as they show a *T*_c_ which is markedly higher compared to *T*_g_’. Another approach is to change the formulation composition in order to obtain a higher critical formulation temperature. To this end, crystalline excipients can be included as bulking agents in combination with amorphous sucrose as stabilizer. Recent publications by Horn et al. and Pansare et al. showed that combinations of crystalline and amorphous excipients allow for aggressive freeze-drying of low concentration protein formulations, while maintaining elegant lyophilisates [14,15]. The ideal ratio of crystalline and amorphous excipients has to be chosen carefully. Crystallization has to be assured which asks for less amorphous excipient content. Yet, the crystalline excipients insufficiently stabilizes the protein [16], which makes a higher amorphous stabilizer necessary. Additional technical problems can arise, e.g., an excessive mannitol content can lead to glass breakage during freeze-drying [17].

Little literature is available on the use of alternative amorphous excipients, such as polysaccharides or polymers, for aggressive cycle development. With their high *T*_g_’ and *T*_c_ as well as potentially protein stabilizing properties due to their amorphous state, such excipients could enable aggressive, short freeze-drying cycles. Larsen et al. recently investigated dextrans, polysaccharides with a *T*_g_’ of −21 °C up to −9 °C depending on their molecular weight, for freeze-drying of lactate dehydrogenase. The dextrans enabled much faster primary drying and provided elegant lyophilisates with good protein stability [18]. In contrast, we found that dextrans were inferior stabilizers for mAbs compared to sucrose [19]. Another excipient of interest is 2-hydroxyproyl-betacyclodextrin (HPBCD), which has a *T*_g_’ similar to dextan 40 kDa [20], and a *T*_c_ of −9 to −6.5 °C [21]. It can be found in approved small molecule parenterals. Furthermore, the use of HPBCD to stabilize proteins in the dried state is described in literature [22]. HPBCD was able to stabilize an antibody during freeze-drying and supercritical fluid drying comparable to trehalose at a protein to sugar ratio of 1:4 (*w/w*) [23,24]. Additionally, HPBCD could provide protein stability during storage at elevated temperatures superior to sucrose [25,26]. The high *T*_g_’ and the protein stabilizing properties of HPBCD might allow for a high *T*_p_ during primary drying while resulting in elegant lyophilisates. To the best of our knowledge, so far, no studies on freeze-drying cycle optimization for HPBD-based mAb formulations are available.

This study aimed to shorten the lyophilization cycle time by using aggressive primary drying conditions for HPBCD-based low concentration mAb formulations (10 mg/mL) containing either pure HPBCD, or combinations with sucrose and polyvinylpyrrolidone (PVP) as excipients, which have all been shown to render amorphous lyophilisates [27,28,29,30]. For a comprehensive study, 50 mg/mL mAb formulations were included as well. The critical formulation temperatures, *T*_g_’ and *T*_c_, were determined and the formulations were freeze-dried with increasingly more aggressive process conditions. Shelf temperatures typically applied for secondary drying were used during primary drying and the hypothesis whether this might enable combining sublimation and desorption of water in one single drying step was tested. The lyophilisates were characterized with regards to cake appearance and structure, reconstitution time, specific surface area, residual moisture, and glass transition temperature of the freeze-dried formulation (*T*_g_), as well as protein stability upon freeze-drying and after storage and compared to a conservatively freeze-dried reference formulation with pure sucrose.

## 2. Materials and Methods

### 2.1. Materials

A F. Hoffmann-La Roche proprietary monoclonal antibody (IgG_1_, pI ~8.2, 149 kDa) was used at 10 mg/mL (low concentration) and 50 mg/mL (high concentration) in 20 mM histidine/histidine-HCl buffer pH 6.0 (Ajinomoto, Tokyo, Japan) with 0.02% polysorbate 20 (Croda International, Snaith, UK). Formulations (F) were prepared with different excipient concentrations as shown in Table S1. F_S_ contained 80 mg/mL sucrose (Ferro Pfanstiehl Company, Mayfield Heights, OH, USA), F_CD_ 80 mg/mL 2‑hydroxypropyl-betacyclodextrin (HPBCD, Roquette, Beinheim, France), F_CD/S_ 56 mg/mL HPBCD and 24 mg/mL sucrose, and F_CD/P/S_ 39.2 mg/mL HPBCD, 16.8 mg/mL polyvinylpyrrolidone K17 (PVP, BASF, Ludwigshafen, Germany), and 24 mg/mL sucrose. Prior to filling, the formulations were filtered through a 0.22 µm PVDF sterile filter unit (Millipore, Bedford, MA, USA). Then, 3.2 mL per formulation were filled into 6 mL TopLyo^®^ vials (Schott, Müllheim, Germany) and partially stoppered with 20 mm Lyo-stoppers D777-1 (DAIKYO Seiko Ltd., Tokyo, Japan).

### 2.2. Freeze-Drying

Freeze-drying was performed using an FTS Lyostar II (FTS Systems Inc., Stone Ridge, NY, USA). Each freeze-drying cycle was performed with one shelf fully loaded. The vials containing the different formulations were distributed randomly over the shelf. To reduce the impact of edge effects on the results, edge vials were excluded from further evaluation. Overall, six different freeze-drying cycles were employed with varying primary and secondary drying conditions as shown in Table 1. Product temperature was determined by three thermocouples in center vials. Chamber pressure (p_c_) was monitored using a Pirani and a capacitance probe. Cycle 0 (C0) represented a typical conservative freeze-drying cycle and was only performed for F_S_. Formulations F_CD_, F_CD/S_, and F_CD/P/S_ at 10 mg/mL mAb were freeze-dried with cycles C1 to C5. F_S_ was freeze-dried with C0 and with C1. C1 and C5 were employed for the 50 mg/mL mAb formulations. At the end of freeze-drying, vials were stoppered at 760 mbar under nitrogen and sealed with aluminum crimp-caps after unloading.

### 2.3. Differential Scanning Calorimetry

*T*_g_’ and *T*_g_ were determined by differential scanning calorimetry according to Haeuser et al. [26]. Measurements were performed using a T_zero_ DSC Q2000 instrument (TA instruments Inc., New Castle, DE, USA). *T*_g_’ and *T*_g_ were determined in triplicates and reported as mean (*T*_g_’, standard deviation < 0.2 °C) and as mean with standard deviation (*T*_g_).

### 2.4. Freeze-Drying Microscopy

Freeze-drying microscopy (FDM) was used to determine *T*_c_ as the temperature of the onset of collapse. Measurements were performed according to Haeuser et al. using a Linkam FDC196 freeze-drying stage (Linkam Scientific, Instruments, Surrey, UK) and a Zeiss Axio Imager.A1 microscope (Carl Zeiss MicroImaging, Göttingn, Germany) at 20-fold magnification [19].

### 2.5. Visual Cake Appearance

Cake appearance of lyophilisates was evaluated by visual inspection. Representative pictures were taken using a camera in front of a black background.

### 2.6. Micro-Computed Tomography

Micro-computed tomography (µ-CT) was performed using an evolved version of the methodology introduced previously [31]. The lyophilisates were analyzed without any further sample preparation though the glass vial with a SkyScan 1272 X-Ray microtomograph (Bruker MicroCT, Kontich, Belgium). Scans were acquired using an acceleration voltage of 40 kV and a beam current of 250 µA. To ensure monochromatic X-rays with enough energy to pass through the glass vial, a 0.5 mm AI filter was applied. The vial was rotated over 360° with a step size of 0.1°. An exposure time of 2388 ms with 10 averages per projection was applied. Projections were reconstructed using the NRecon software (Bruker, Kontich, Belgium) to obtain an image stack of tomographs.

### 2.7. Reconstitution Time

For reconstitution, 3.0 or 2.9 mL of water for injection were added to the 10 and 50 mg/mL mAb formulations, respectively, using a 5 mL disposable syringe equipped with a 21 G needle. Reconstitution time was determined in triplicates as described previously and reported as mean with standard deviation [19].

### 2.8. Specific Surface Area

Specific surface area was determined in triplicates according to Brunauer–Emmett–Teller (BET) using the Quadrasorb evo surface area and pore size analyzer (Quantachrome, Odelzhausen, Germany) with Krypton as adsorbate. Analysis was performed in a 9 mm bulb sample cell filled with at least 100 mg lyophilisate. Prior to analysis, the samples were degassed overnight under vacuum at 40 °C and overlaid with Nitrogen. Krypton adsorption was determined for nine measuring points at 77 K over a pressure range of 0.05 to 0.25 mbar. Specific surface area was determined by fitting the data points using the BET equation and reported as mean with standard deviation.

### 2.9. Residual Moisture

Residual moisture was determined in triplicates according to Haeuser et al. using a C30 Coulometric Karl Fischer titrator (Mettler Toledo, Greifensee, Switzerland). Residual moisture was reported as mean with standard deviation [19].

### 2.10. Size-Exclusion Chromatography

Stability of the mAb was analyzed by size-exclusion high-performance liquid chromatography (SE-HPLC) using an Alliance e2695 HPLC instrument (Waters Corporation, Milford, MA, USA) equipped with a 2487 UV/visible detector (Waters Corporation, Milford, MA, USA). Samples were held at 5 °C and the column temperature was set to 25 °C. Then, 50 mg/mL mAb formulations were diluted to 10 mg/mL with formulation buffer, 10 mg/mL mAb formulations were analyzed without further preparation. A total of 10 µL of the sample was injected on a TSKG3000SWxl, 7.8 × 300 mm column (Tosoh Bioscience, Stuttgart, Germany) and eluted over 30 min with a 0.2 M K_2_HPO_4_/KH_2_PO_4_ and 0.25 M KCl of pH 6.2. Signal was detected as UV absorbance at 280 nm. Data processing was done using the Empower 3 Chromatography Data System software v. 4 (Waters Corporation, Milford, MA, USA) and monomer content was reported as percentage of total peak area.

## 3. Results

### 3.1. Thermal Properties of the Liquid Formulations

To assess the critical *T*_p_, *T*_g_’, and *T*_c_ were determined for 10 and 50 mg/mL mAb formulations (Table 2). The low concentration reference formulation (F_s_) had a much lower *T*_g_’ of −29.5 °C compared to the other formulations. Similar *T*_g_’ values were obtained for F_CD/P/S_ (−19.2 °C) and F_CD/S_ (−18.5 °C), which were both more than 10 °C above the *T*_g_’ of F_s_. The highest *T*_g_’ values among all formulations were obtained for pure HPBCD formulations with −10.9 and −8.3 °C for 10 and 50 mg/mL mAb, respectively. In contrast to the other formulations where *T*_c_ was slightly above *T*_g_’, F_S_ had a *T*_c_ of −31.0 °C, which was slightly lower than its *T*_g_’. Generally, formulations with 50 mg/mL mAb showed markedly higher *T*_g_’ and *T*_c_ values compared to 10 mg/mL mAb formulations. The impact of protein concentration on *T*_g_’ was more pronounced the lower the *T*_g_’ of the formulations compared to each other. This means that the *T*_g_’ of F_S_ increased by 4.1 °C from −29.5 to −25.4 °C when increasing the mAb concentration from 10 to 50 mg/mL compared to an increase of only 2.6 °C for F_CD_ for example. In addition, for F_CD_, F_CD/P/S_, and F_CD/S_ with 50 mg/mL mAb, the difference between *T*_g_’ and *T*_c_ was at least twice that of 10 mg/mL mAb formulations. For instance, at low mAb concentration, *T*_c_ of F_CD/P/S_ was 0.7 °C and for high mAb concentrations 1.9 °C above the *T*_g_’.

### 3.2. Impact of Freeze-Drying Parameters on T_p_ and Primary Drying Time

**Figure 1:**
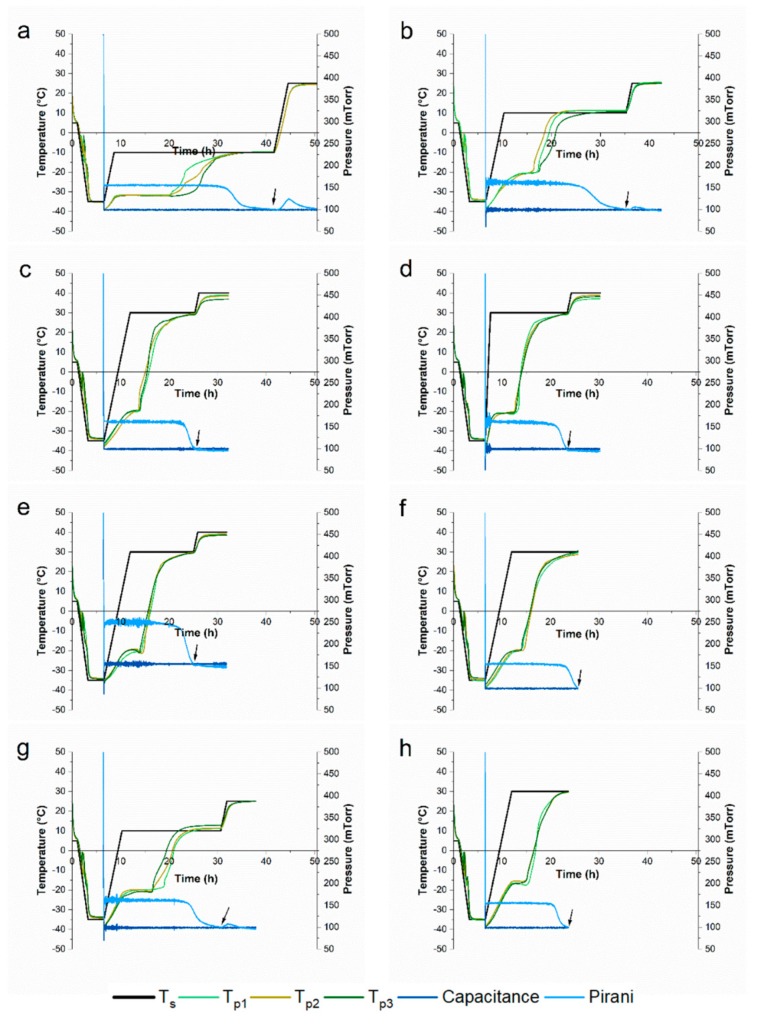
Freeze-drying cycles. Process monitoring data of (**a**–**f**) 10 mg/mL mAb formulations and of (**g**–**h**) 50 mg/mL monoclonal antibody (mAb) formulations. (**a**) The conservative cycle (C0), (**b**,**g**) C1, (**c**) C2, (**d**) C3, (**e**) C4, and (**f**,**h**) C5. The arrow indicates end of primary drying. *T*_s_ = shelf temperature; *T*_p_ = product temperature determined by thermocouples.

As reference, a conservative freeze-drying cycle (C0), typically used for sucrose-based formulations, with *p*_c_ = 100 mTorr and *T*_s_ = −10 °C during primary drying was performed for F_S_. Freeze-drying with C0 resulted in a *T*_p_ in the steady state of primary drying of ~−32 °C and a primary drying time of ~35 h with 50.5 h total cycle time (Figure 1a). As a start, *T*_s_ during primary drying was increased to +10 °C (C1), showing a much shorter steady state during primary drying with a mean *T*_p_ of ~−21 °C, as shown in Figure 1b. Primary drying time, i.e., total cycle time, was shortened by 6 h, corresponding to a reduction of 12%. To further shorten the freeze-drying cycle time, F_CD_, F_CD/P/S_, and F_CD/S_ were lyophilized with a T_s_ during primary drying of +30 °C and a *T*_s_ of +40 °C during secondary drying (C2). Figure 1c shows that increasing *T*_s_ from +10 to +30 °C resulted in a freeze-drying cycle with a very short steady state phase at a *T*_p_ of ~−20 °C. Ultimately, primary drying time was reduced to ~19 h. Using a low ramp rate (0.2 °C/min) from freezing to primary drying and the high temperature difference of 65 °C to overcome, the ramping step contributed with ~17% markedly to the total cycle time of 32.2 h. Hence, C3 was performed using a ramp rate from freezing to primary drying of 1 °C/min. Consequently, primary drying time was reduced by another 2 h and showed a slightly more pronounced steady sublimation phase compared to C2 (Figure 1d). Another approach to accelerate primary drying is to increase the pressure. To this end, primary and secondary drying in C4 were performed at a p_c_ of 155 mTorr (Figure 1e). Increasing p_c_ only slightly increased *T*_p_ to ~−19.0 °C, but did not result in a shorter primary drying phase compared to C2. The aggressive lyophilization cycles C2–C4 showed no increase in the Pirani signal (Figure 1c–e) during secondary drying, indicating that the desorption phase was already finished at the end of primary drying. Hence, a final single-step freeze-drying cycle was used without a secondary drying step, leading to a total cycle time of ~25 h (Figure 1f). This resulted in a 50% reduction of total cycle time compared to the conservative cycle. Highly concentrated mAb formulations were freeze-dried using only two cycles, the initial C1 and the final C5. In general, *T*_p_ in the steady state during primary drying was slightly higher compared to 10 mg/mL mAb formulations, with a *T*_p_ of ~−20 °C compared to ~−21 °C during C1 and a *T*_p_ of ~−17 °C compared to ~−20 °C during C5. Consequently, primary drying was completed earlier, i.e., after 24 h for C1 and after 17 h for C5 (Figure 1g−h).

### 3.3. Cake Appearance and Structure

**Figure 2:**
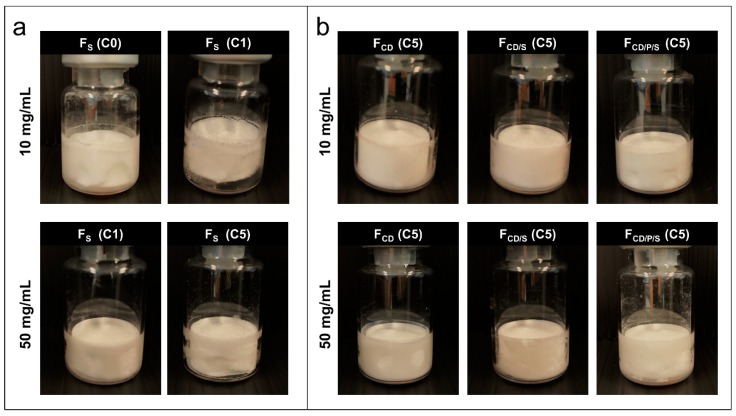
Cake appearance. Representative lyophilisates for 10 mg/mL formulations (upper row) and 50 mg/mL formulations (lower row). (**a**) Different cake appearances for reference formulation (F_S_) depending on freeze-drying cycle and (**b**) cake appearance obtained for F_CD_, F_CD/S_, and F_CD/P/S_ exemplarily shown for C5.

**Figure 3:**
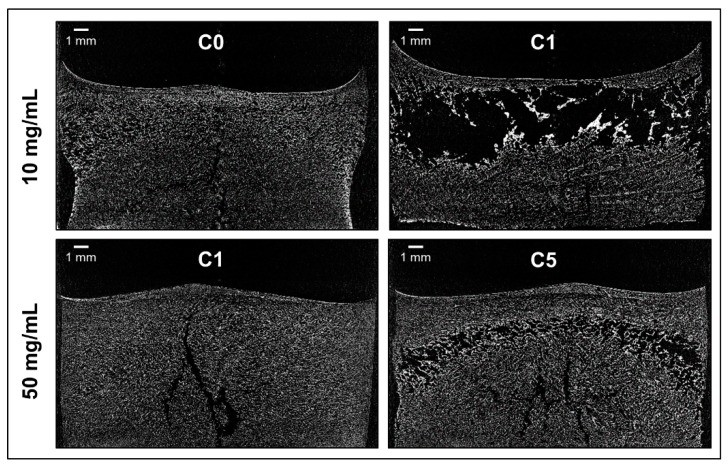
Internal cake structure of F_S_. Representative micro-computed tomography (µ-CT) images for 10 and 50 mg/mL formulations freeze-dried with different lyophilization cycles.

**Figure 4:**
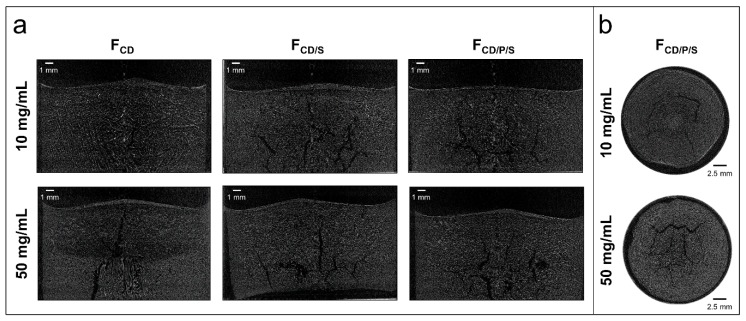
Internal cake structure. Representative µ-CT images for 10 and 50 mg/mL formulations freeze-dried with C5 showing (**a**) internal cake structure through a vertical cross section of F_CD_, F_CD/S_, and F_CD/P/S_, and (**b**) cake structure at the bottom of the vial through a horizontal cross section of F_CD/P/S_.

Cake appearance was investigated visually as well as by µ-CT to obtain a comprehensive evaluation of the structure. In preliminary experiments, better, less brittle or cracked cake appearance was obtained for all formulations when freeze-dried in TopLyo^®^ vials compared to Fiolax^®^ vials. Therefore, only TopLyo^®^ vials were used for all experiments. F_S_ with 10 mg/mL mAb showed major dents when freeze-dried with the reference cycle (C0) (Figure 2a and Figure 3). The increase of *T*_s_ during primary drying to +10 °C resulted in collapse (Figure 2a). µ-CT images of the internal structure revealed a total loss of cake structure in the upper half of the lyophilisate (Figure 3). F_CD_, F_CD/P/S_, and F_CD/S_ at 10 mg/mL mAb resulted in elegant lyophilisates throughout all lyophilization cycles C1–C5, with only minor dents at the bottom of the vial for F_CD/P/S_ as shown in Figure 2b_._ These dents were slightly more pronounced in C4. Differences in the internal cake structure between the formulations were detected by µ‑CT. Pure HPBCD formulations rendered homogenous cakes. Some cracks formed in F_CD/S_ and F_CD/P/S_ formulations (Figure 4).

At 50 mg/mL mAb F_S_ resulted in pharmaceutically elegant lyophilisates (Figure 2) with internal cracks, as shown in Figure 3, when freeze-dried with C1. At more aggressive conditions with a *T*_s_ of +30 °C (C5), major dents were observed by visual inspection (Figure 2) and µ-CT revealed a collapsed internal cake structure in the upper half of the lyophilisate. F_CD_, F_CD/P/S_, and F_CD/S_ at high mAb concentration resulted in visually elegant lyophilisates for both cycles C1 and C5. Interestingly, the internal cake structure of F_CD_ at 50 mg/mL mAb was different compared to the 10 mg/mL mAb formulation. At higher mAb concentration, the cake showed a large lamellar like structure in the middle-bottom region and a smaller spherical structure, similar to 10 mg/mL formulations, in the upper half of the lyophilisate (Figure 4). To elucidate on the root cause of this observation was beyond the scope of this study.

### 3.4. Other Product Quality Attributes

**Figure 5:**
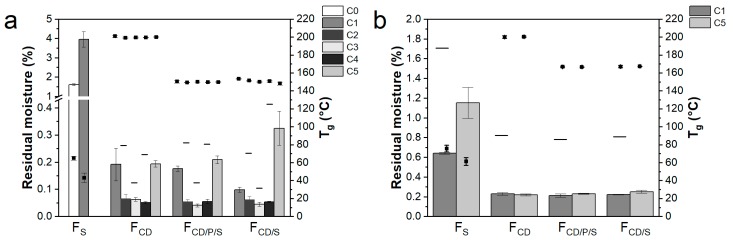
Residual moisture and *T*_g_. Data is given for (**a**) 10 mg/mL mAb formulations and (**b**) 50 mg/mL mAb formulations for different formulations and freeze-drying cycle conditions. Residual moisture levels are shown as bars and lines. Lines show residual moisture level of vials stoppered after primary drying. Squares show *T*_g_ values of the lyophilisates. Values are means (*n* = 3) ± standard deviation.

Other important product quality attributes to study when optimizing lyophilization processes are reconstitution time, specific surface area, residual moisture, and *T*_g_. All 10 mg/mL mAb formulations reconstituted fast (<60 s) without an effect of the lyophilization cycle employed (Figure S1a). For 50 mg/mL mAb formulations, reconstitution was generally slower and took at least twice as long. Reconstitution of F_S_ at 50 mg/mL mAb took the longest with ~80 s compared to F_CD_, F_CD/P/S_, and F_CD/S_, independent of the cycle (Figure S1b). In general, no major differences were found in specific surface area for the different excipient combinations and freeze-drying cycles employed for both 10 and 50 mg/mL formulations (Figure S2). Only F_S_ with 10 mg/mL mAb showed a markedly lower specific surface area when freeze-dried with C1 compared to C0, indicating collapse. More pronounced differences were observed for the residual moisture as shown in Figure 5. For 10 mg/mL mAb formulations (Figure 5a), residual moisture of F_CD_, F_CD/P/S_, F_CD/S_ was less than 0.5%, with marginal differences amongst the different formulations. In contrast, F_S_ had a residual moisture of 1.2% when freeze-dried with the conservative cycle C0. When freeze-dried with the more aggressive cycle C1, residual moisture levels increased to 4.1%. For C2 to C4 (or C1 for 50 mg/mL mAb formulations), some vials were stoppered at the end of primary drying to evaluate the remaining moisture content at the end of primary drying. Residual moisture of these stoppered samples varied more between the different formulations but without a clear trend. Products from C3 showed the least variation in residual moisture at the end of primary drying. Overall, already after primary drying residual moisture was below 0.5%, supporting the observations for the Pirani signal (Figure 1c–e).

Residual moisture of F_S_ with 50 mg/mL mAb was generally higher compared to F_CD_, F_CD/P/S_, and F_CD/S_ (Figure 5b). C1 resulted in a residual moisture for F_S_ of 0.64%, while freeze-drying with C5 led to a residual moisture of 1.1%. F_CD_, F_CD/P/S_, and F_CD/S_ with 50 mg/mL mAb showed low residual moisture levels of ~0.2%, identical for C1 and C5 and similar to the values for the 10 mg/mL mAb formulations.

The varying residual moisture levels of 10 mg/mL F_S_ when freeze-dried with different cycles were reflected in different *T*_g_ values. While C0 resulted in lyophilisates with a *T*_g_ of 65 °C, the *T*_g_ of the collapsed F_S_ was considerably lower with 43.2 °C. *T*_g_ values of F_CD_, F_CD/P/S_, and F_CD/S_ were much higher than compared to F_S_ and were comparable for all cycles employed. *T*_g_ of F_CD/P/S_ and F_CD/S_ showed a similar *T*_g_ at ~150 °C, while F_CD_ had a *T*_g_ of ~200 °C. Corresponding to the *T*_g_’ of the liquid formulations, *T*_g_ was generally higher for 50 mg/mL mAb formulations with differences in *T*_g_ for F_S_ when freeze-dried with C1 or C5.

### 3.5. Protein Stability

**Figure 6:**
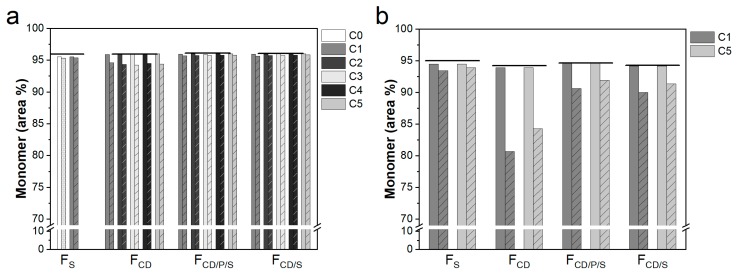
Protein stability by size-exclusion high-performance liquid chromatography (SE-HPLC). Percentage of remaining monomer for (**a**) 10 mg/mL mAb formulations and (**b**) 50 mg/mL mAb for different formulations and freeze-drying cycle conditions. Bars without pattern show monomer content directly after freeze-drying and bars with pattern show monomer content after storage at 40 °C for 3 months. Lines show the monomer content of the liquid formulation prior to freeze-drying.

Protein stability was investigated as remaining monomer content by SE-HPLC directly after freeze-drying as well as after storage at 40 °C for 3 months (Figure 6). All formulations demonstrated sufficient cryo- and lyoprotection for both mAb concentrations, independent of the freeze-drying cycle employed. Good storage stability was obtained for the 10 mg/mL mAb F_S_ reference formulation after storage at 40 °C for 3 months when freeze-dried with the conservative as well as the more aggressive cycle C1. Stability of F_CD_, F_CD/P/S_, and F_CD/S_ at 10 mg/mL mAb was comparable to F_S_. F_S_, F_CD/S_, and F_CD/P/S_ showed a marginally but consistent decrease in monomer content of 0.1%–0.2% after storage (Figure 4a), which went along with an increase in high molecular weight species (data not shown). F_CD_ lyophilisates were less stable during storage (~1.5% loss of monomer) throughout all freeze-drying cycles. While 50 mg/mL mAb formulations also demonstrated good protein stability during freeze-drying, they generally showed a higher loss of monomer during storage. A 1% decrease in monomer content was observed for F_s_ after storage for both freeze-drying cycles. The remaining monomer content after storage was much lower for F_CD_ with levels of 80.7% and 84.3% for C1 and C5, respectively. Better and similar mAb stability was obtained for F_CD/S_ and F_CD/P/S_, with a loss of monomer content of 4.1% for C1 and 2.7% for C5. Interestingly, for F_CD_, F_CD/S_, and F_CD/P/S_ with 50 mg/mL mAb stability was generally slightly improved when lyophilized with C5 compared to C1.

In an additional experiment, we investigated whether the aggressive freeze-drying cycle that we used might result in so-called overdrying of the mAb (i.e., ~0.2% residual moisture). Therefore, defined residual moisture levels were prepared for 10 mg/mL formulations by spiking of the lyophilisates with water droplets. Residual moisture dependent protein stability was subsequently investigated. Our data in Figure S3 shows similar protein stability for lyophilisates with a residual moisture of 0.2%, 0.5%, 1%, or 2% after 3 months at 40 °C.

## 4. Discussion

The aim of this study was to develop an aggressive, thus short freeze-drying cycle for amorphous formulations with a higher *T*_g_’ and *T*_c_ compared to pure sucrose that results in elegant cakes, low residual moisture levels, fast reconstitution time, and has no negative impact on protein stability. A sucrose-based formulation was included for reference purpose to (i) compare cycle time and product quality attributes with traditional sucrose-based formulations and (ii) demonstrate the limits of pure sucrose with regards to shortening freeze-drying.

### 4.1. Correlation Between T_g_’, T_c_, T_p_, and Cake Appearance

In a first step during formulation and freeze-drying cycle development, it is essential to determine *T*_g_’ and *T*_c_ of the liquid formulation, as they are indicative for the *T*_p_ which should not be exceeded during primary drying to avoid collapse. *T*_c_ is commonly considered to be the more accurate predictor and is typically few degrees above *T*_g_’ [32,33]. For formulations with low protein concentrations, *T*_g_’ and *T*_c_ may be used interchangeably. In fact, in our study *T*_c_ of F_S_ was even slightly lower than the *T*_g_’. This is consistent with data previously reported by Colandene et al. for sucrose-based 10 mg/mL protein formulations [11]. At higher protein concentrations, *T*_c_ is markedly higher than *T*_g_’ and the difference (Δ*T*) increases with higher protein concentration. For a 50 mg/mL mAb formulation with 7–8% disaccharide, a Δ*T* of 5 °C was reported and Depaz et al. found a *T*_c_ 14 °C above the *T*_g_’ for a 100 mg/mL mAb formulation [13,15]. This is in line with our results of a Δ*T* of 5°C for the 50 mg/mL pure sucrose formulation. For formulations containing excipients which have a high *T*_g_’ themselves, the effect of protein concentration on Δ*T* is less pronounced. For F_CD/S_ and F_CD/P/S_ both 10 mg/mL formulations showed very similar *T*_g_’ values, but their *T*_c_ differed by 2.6 °C. This directly translated into different cake appearance when freeze-dried with the same cycles with *T*_p_ close to *T*_g_’ (C1–C5). *T*_c_ depends on several factors such as the solid concentration as well as sublimation rate. Thus, collapse in the vial might occur at slightly higher temperatures during freeze-drying than the *T*_c_ determined by FDM [33]. Greco et al. used optical coherence tomography to determine *T*_c_ of a 5% sucrose solution in the vial during freeze-drying and found it to be 3 °C above that temperature determined by FDM [34]. This is in line with the cake appearance observed in our study. F_s_ at low protein concentration showed major dents when freeze-dried with the conservative lyophilization cycle, where *T*_p_ was close to *T*_c_. Collapse occurred only when *T*_p_ was much higher than *T*_c_, as it was the case for C1. In terms of internal cake structure, µ-CT analysis showed minor cracks in most lyophilisates independent of the cycle employed. Patel et al. suggested that cracks should not be considered cake defects as they are only process artefacts which are not detrimental to product quality [8]. In fact, internal cracks have been found to be a result of relieved stress during secondary drying, when unfrozen water is removed [35,36]. Lam et al. suggested that the formation of splitted cakes might be linked to a complex interplay of events occurring during the freezing step. They also reported that the occurrence of cracks is highly variable, and observed that cracks can be present in lyophilisates that look pharmaceutically elegant from the outside [37], in line with our observations. More important are internal defects such as partial collapse, which was revealed by µ-CT for e.g., 50 mg/mL F_S_ when freeze-dried with C5. This highlighted in addition that the dents observed by visual inspection were truly correlated to the onset of collapse.

### 4.2. Impact of Process Parameters

It is well established that an increase of *T*_s_ during primary drying reduces cycle time [3]. Correspondingly, as we increased *T*_s_ during primary drying from −10 °C to ultimately +30 °C, we substantially shortened primary drying by 48%. Additional elimination of secondary drying shortened the overall cycle time by in total 50%. Although both increases in *T*_s_, from −10 to +10 °C (C1) and from +10 to +30 °C (C2), strongly impacted process time, the latter only marginally increased *T*_p_. Similar to our results, Depaz et al. reported that increasing *T*_s_ from −30 to 0 °C resulted in a marked increase of *T*_p_ for a 25 mg/mL mAb formulation, and led to a brief steady sublimation phase, whereas a further increase of *T*_s_ to +15 °C further shortened the steady sublimation phase without impacting *T*_p_ [13]. In addition, aggressive primary drying temperatures resulted in a steeper drop of the Pirani signal. A fast drop of the Pirani signal implies a homogenously dried batch [38], which is desirable, in particular if aiming to omit secondary drying. Greater batch homogeneity was additionally demonstrated by smaller variations within the temperature probes for aggressive lyophilization conditions. When aiming for aggressive freeze-drying cycles at high *T*_s_, the ramping step contributes markedly to the total cycle time. Typically, ramp rates of less than 1.0 °C/min are applied, but faster ramp rates of e.g., 1 °C/min are also suitable. Horn et al. found no negative impact when increasing the ramp rate into primary drying from 0.5 °C to 1 °C/min [14]. Ohio et al. demonstrated that faster ramp rates might result in even better cake appearance for high *T*_s_ conditions during primary drying compared to very slow ramp rates [39,40]. In contrast, Pansare et al. reported more pronounced shrinkage for lyophilisates that were freeze-dried using a ramp rate of 0.5 °C/min compared to 0.1 °C/min [15]. In the present study, although product quality attributes like residual moisture, specific surface area, reconstitution time, and qualitative internal cake structure did not show any differences for C3 compared to C2, we visually observed some lifted cakes after freeze-drying. However, the time point when lifting occurred remained unknown. In the light of these observations and a potential gain of only 2 h in overall cycle time, we decided to stick with the low ramp rates for the following cycles C4 and C5. In general, selection of the optimal p_c_ is a balance between batch homogeneity and prevention of collapse or meltback [3,41]. Previous studies demonstrated an increase of p_c_ to be advantageous for shorter freeze-drying cycles, leading to higher *T*_p_ and thus shorter cycle times [14]. However, in the present study, although *T*_p_ was slightly higher in C4 compared to C2, no impact on primary drying time was observed when using a pressure of 155 mTorr compared to 100 mTorr. At 155 mTorr, *T*_p_ showed a slight drop after a short steady state (C4) prior to its increase. It has been speculated that this might be indicative for micro-collapse [13,15]. For all aggressive cycles (C2–C4), the Pirani signal indicated end of overall drying, i.e., sublimation and desorption, already at the end of primary drying. Finally, F_S_, F_CD/P/S_, and F_CD/S_ allowed for a single-step freeze-drying, resulting in comparable product quality attributes to a conservative cycle. Pansare et al. also applied a single step freeze-drying for amorphous formulations, but their disaccharide-based formulations resulted in product shrinkage and partial collapse for formulations with 25 mg/mL or less protein and minor shrinkage at 50 mg/mL. Addition of a crystalline excipient was necessary to obtain elegant lyophilisates, which however led to slightly higher aggregation rates compared to purely amorphous formulations during storage [15].

### 4.3. Protein Stability

When optimizing freeze-drying processes, it is of utmost importance to ensure protein stability. In our study, no negative impact of the process parameters on protein stability, including *T*_s_ of +30 °C during primary drying and +40 °C during secondary drying was observed for any formulation, which is in line with previous studies [13,14,15]. Tang and Pikal reported that dried protein formulations will not undergo denaturation at temperatures up to 100 °C for short periods [3]. In terms of storage stability, collapse is often of concern as it may result in higher residual moisture and lower *T*_g_. Various studies report that collapse itself did not reduce protein stability during storage [6,7]. On the contrary, Lueckel et al. demonstrated that collapse resulted in increased aggregation of an IL-6 lyophilisate in a sucrose/glycine formulation after storage [5]. Correspondingly, Passot et al. reported 25% loss of activity for lyophilized toxins in a PVP/sucrose or PVP/mannitol matrix after 6 months of storage, when freeze-dried with a *T*_p_ above *T*_g_’ during primary drying [4]. The results of the present study showed good and comparable storage stability for F_CD/P/S_ and F_CD/S_. Collapse did not impact protein stability of F_S_ when stored at 40 °C for 3 months. However, we previously found substantial protein degradation for a collapsed 10 mg/mL sucrose-based formulation when stored at 40 °C for 6 months or longer [26].

At 50 mg/mL mAb, all formulations showed a higher loss of monomer compared to the 10 mg/mL formulation after 3 months at 40 °C. The increase in aggregates was more pronounced for F_CD/S_, F_CD/P/S_, and F_CD_ compared to F_s_. Within this study the excipient solid content was kept constant at 80 mg/mL for both low and high mAb concentrations. Thus, for the 50 mg/mL mAb formulations the excipient to protein ratio was too low to adequately protect the protein. Similarly, Lewis et al. reported good mAb stability when formulated at 5 mg/mL but observed protein aggregation at 20 mg/mL in 25 mg/mL sucrose lyophilisates [42]. Their excipient to protein ratios of 4:1 and 1.2:1 (*w/w*) are close to our 7:1 and 1.6:1 ratio for 10 and 50 mg/mL mAb, respectively. Cleland et al. reported that a molar excipient to protein ratio of at least 360:1 is necessary in order to ensure good protein stability at 40 °C for 3 months [43]. This ratio was easily exceeded for the 10 mg/mL mAb formulations in our study. For the 50 mg/mL mAb formulations, only F_S_ (695:1) was at this level, whereas F_CD/S_, F_CD/P/S_, and F_CD_ reflected ratios of only 290:1, 321:1, and 163:1, respectively. Interestingly, we achieved a better storage stability for 50 mg/mL mAb formulations freeze-dried with C5 compared to C1, although no differences were observed in the physico-chemical product quality attributes. To elucidate the influence of excipient to protein ratio and process parameters on formulations with a mAb concentration above 50 mg/mL was beyond the scope of the present study.

In summary, a binary combination of HPBCD and sucrose at a 7:3 ratio (*w/w*) provides a highly attractive amorphous formulation. This formulation allows for a 50% cycle time reduction compared to the conventional reference cycle through single-step freeze-drying of low concentration biopharmaceuticals, resulting in elegant lyophilisates with short reconstitution times, low residual moisture, high *T*_g_, and good protein stability during storage. Moreover, the actual effect on cycle time might be even more pronounced as the reference cycle with sucrose formulations at a mAb concentration of 10 mg/mL would require even lower primary drying in order to eliminate dents.

## 5. Conclusions

Within the present study we demonstrated that scientists can be very aggressive during freeze-drying, using HPBCD-based formulations in combination with sucrose or PVP/sucrose. We were able to reduce cycle time by 50%, obtaining pharmaceutical elegant lyophilisates for pure HPBCD and HPBCD/sucrose, while HPBCD/PVP/sucrose showed minor dents. All other product quality attributes were similar, acceptable, and comparable to the conservatively freeze-dried sucrose formulation. Protein stability was ensured for all formulations upon freeze-drying and combinations of HPBCD/sucrose and HPBCD/PVP/sucrose at 10 mg/mL mAb provided good stability during storage at 40 °C for 3 months. We believe that the proposed excipient combinations can be applied for higher concentrated protein formulations as well by adjustment of excipient to protein ratio. We conclude that the proposed single-step freeze-drying cycle using a binary mixture of HPBCD/sucrose has the potential to significantly reduce costs of goods due to more efficient freeze-drying, while maintaining elegant lyophilisates and ensuring protein stability.

## Figures and Tables

**Table 1:** Overview of process parameters of the different freeze-drying cycles employed. All cycles had a loading step of 60 min at 5 °C prior to freezing. End of primary drying time was determined as ∆p_c_ ≤ 1 mTorr between Pirani and capacitance probe.

Cycle	Freezing	Primary Drying			Secondary Drying			
		Ramp (°C/min)	*T*_s_ (°C)	p_c_ (mTorr)	Ramp (°C/min)	*T*_s_ (°C)	Hold Time (min)	Pressure (mTorr)
0	Ramp 0.3 °C/min Temp. −35 °C Hold 180 min	0.2	−10	100	0.2	+25	360	100
1		0.2	+10	100	0.2	+25	360	100
2		0.2	+30	100	0.2	+40	360	100
3		1.0	+30	100	0.2	+40	360	100
4		0.2	+30	155	0.2	+40	360	155
5		0.2	+30	100	-			

*T*_s_: shelf temperature, p_c_: chamber pressure.

**Table 2:** Critical formulation temperatures. Glass transition temperature (*T*_g_’) and onset of collapse temperature (*T*_c_) of the liquid formulations.

Formulation	mAb Concentration(mg/mL)	*T*_g_’ (°C)	*T*_c_ (°C)
F_S_	10	−29.5	−31.0
	50	−25.4	−20.4
F_CD_	10	−10.9	−10.1
	50	−8.3	−6.3
F_CD/P/S_	10	−19.2	−18.5
	50	−16.5	−14.6
F_CD/S_	10	−18.6	−16.0
	50	−15.2	−10.9

## Supplementary Materials

The following are available online at https://www.mdpi.com/1999-4923/11/11/616/s1, Table S1: Formulation composition. Figure S1: Reconstitution time of lyophilisates. Figure S2: Specific surface area of lyophilisates.

## References

[1] Gervasi V., Agnol R.D., Cullen S., McCoy T., Vucen S., Crean A. (2018). Parenteral protein formulations: An overview of approved products within the European Union. Eur. J. Pharm. Biopharm..

[2] Constantino H.R., Constantino H.R., Pikal M.J. (2004). Excipients for use in lyophilized pharmaceutical peptide, protein and other bioproducts. Lyophilization of Biopharmaceuticals.

[3] Tang X., Pikal M.J. (2004). Design of freeze-drying processes for pharmaceuticals: Practical advice. Pharm. Res..

[4] Passot S., Fonseca F., Barbouche N., Marin M., Alarcon-Lorca M., Rolland D., Rapaud M. (2007). Effect of Product Temperature During Primary Drying on the Long-Term Stability of Lyophilized Proteins. Pharm. Dev. Technol..

[5] Lueckel B., Helk B., Bodmer D., Leuenberger H. (1998). Effects of Formulation and Process Variables on the Aggregation of Freeze-Dried Interleukin-6 (IL-6) After Lyophilization and on Storage. Pharm. Dev. Technol..

[6] Wang D.Q., Hey J.M., Nail S.L. (2004). Effect of collapse on the stability of freeze-dried recombinant factor VIII and alpha-amylase. J. Pharm. Sci..

[7] Schersch K., Betz O., Garidel P., Muehlau S., Bassarab S., Winter G. (2012). Systematic Investigation of the Effect of Lyophilizate Collapse on Pharmaceutically Relevant Proteins, Part 2: Stability During Storage at Elevated Temperatures. J. Pharm. Sci..

[8] Patel S.M., Nail S.L., Pikal M.J., Geidobler R., Winter G., Hawe A., Davagnino J., Gupta S.R. (2017). Lyophilized Drug Product Cake Appearance: What Is Acceptable?. J. Pharm. Sci..

[9] Horn J., Friess W. (2018). Detection of Collapse and Crystallization of Saccharide, Protein, and Mannitol Formulations by Optical Fibers in Lyophilization. Front. Chem..

[10] Gitter J.H., Geidobler R., Presser I., Winter G. (2018). Significant Drying Time Reduction Using Microwave-Assisted Freeze-Drying for a Monoclonal Antibody. J. Pharm. Sci..

[11] Colandene J.D., Maldonado L.M., Creagh A.T., Vrettos J.S., Goad K.G., Spitznagel T.M. (2007). Lyophilization Cycle Development for a High-Concentration Monoclonal Antibody Formulation Lacking a Crystalline Bulking Agent. J. Pharm. Sci..

[12] Bjelošević M., Seljak K.B., Trstenjak U., Logar M., Brus B., Grabnar P.A. (2018). Aggressive conditions during primary drying as a contemporary approach to optimise freeze-drying cycles of biopharmaceuticals. Eur. J. Pharm. Sci..

[13] Depaz R.A., Pansare S., Patel S.M. (2016). Freeze-Drying Above the Glass Transition Temperature in Amorphous Protein Formulations While Maintaining Product Quality and Improving Process Efficiency. J. Pharm. Sci..

[14] Horn J., Schanda J., Friess W. (2018). Impact of fast and conservative freeze-drying on product quality of protein-mannitol-sucrose-glycerol lyophilizates. Eur. J. Pharm. Biopharm..

[15] Pansare S.K., Patel S.M. (2019). Lyophilization Process Design and Development: A Single-Step Drying Approach. J. Pharm. Sci..

[16] Izutsu K., Yoshioka S., Terao T. (1993). Decreased Protein-Stabilizing Effects of Cryoprotectants Due to Crystallization. Pharm. Res..

[17] Williams N.A., Dean T. (1991). Vial breakage by frozen mannitol solutions: Correlation with thermal characteristics and effect of stereoisomerism, additives, and vial configuration. PDA J. Pharm. Sci. Technol..

[18] Larsen B.S., Skytte J., Svagan A.J., Meng-Lund H., Grohganz H., Lobmann K. (2019). Using dextran of different molecular weights to achieve faster freeze-drying and improved storage stability of lactate dehydrogenase. Pharm. Dev. Technol..

[19] Haeuser C., Goldbach P., Huwyler J., Friess W., Allmendinger A. (2019). Impact of dextran on thermal properties, product quality attributes, and monoclonal antibody stability in freeze-dried formulations. Eur. J. Pharm. Biopharm..

[20] Wong J., Kipp J.E., Miller R.L., Nair L.M., Ray G.J. (2014). Mechanism of 2-hydropropyl-*β*-cyclodextrin in the stabilization of frozen formulations. Eur. J. Pharm. Sci..

[21] Meister E., Šaši S., Gieseler H. (2009). Freeze-Dry Microscopy: Impact of Nucleation Temperature and Excipient Concentration on Collapse Temperature Data. AAPS PharmSciTech.

[22] Serno T., Geidobler R., Winter G. (2011). Protein stabilization by cyclodextrins in the liquid and dried state. Adv. Drug Deliv. Rev..

[23] Jovanović N., Bouchard A., Hofland G.W., Witkamp G.-J., Crommelin D.J., Jiskoot W. (2008). Stabilization of IgG by supercritical fluid drying: Optimization of formulation and process parameters. Eur. J. Pharm. Biopharm..

[24] Faghihi H., Merrikhihaghi S., Najafabadi A.R., Ramezani V., Sardari S., Vatanara A. (2016). A comparative study to evaluate the effect of different carbohydrates on the stability of immunoglobulin G during lyophilization and following storage. Pharm. Sci..

[25] Ressing M.E., Jiskoot W., Talsma H., Van Ingen C.W., Beuvery E.C., Crommelin D.J. (1992). The influence of sucrose, dextran, and hydroxypropyl-*β*-cyclodextrin as lyoprotectants for a freeze-dried mouse IgG_2a_ monoclonal antibody (MN12). Pharm. Res..

[26] Haeuser C., Goldbach P., Huwyler J., Friess W., Allmendinger A. (2019). Excipients for room temperature stable freeze-dried monoclonal antibody formulations. J. Pharm. Sci..

[27] Nunes C., Mahendrasingam A., Suryanarayanan R. (2005). Quantification of Crystallinity in Substantially Amorphous Materials by Synchrotron X-ray Powder Diffractometry. Pharm. Res..

[28] Padilla A.M., Ivanisevic I., Yang Y., Engers D., Bogner R.H., Pikal M.J. (2011). The Study of Phase Separation in Amorphous Freeze-Dried Systems. Part I: Raman Mapping and Computational Analysis of XRPD Data in Model Polymer Systems. J. Pharm. Sci..

[29] Bandi N., Wei W., Roberts C.B., Kotra L.P., Kompella U.B. (2004). Preparation of budesonide- and indomethacin-hydroxypropyl-*β*-cyclodextrin (HPBCD) complexes using a single-step, organic-solvent-free supercritical fluid process. Eur. J. Pharm. Sci..

[30] Geidobler R. (2014). Cyclodextrins as Excipients in Drying of Proteins and Controlled Ice Nucleation in Freeze-Drying. Ph.D. Thesis.

[31] Haeuser C., Goldbach P., Huwyler J., Friess W., Allmendinger A. (2018). Imaging Techniques to Characterize Cake Appearance of Freeze-Dried Products. J. Pharm. Sci..

[32] Meister E., Gieseler H. (2009). Freeze-Dry Microscopy of Protein/Sugar Mixtures: Drying Behavior, Interpretation of Collapse Temperatures and a Comparison to Corresponding Glass Transition Data. J. Pharm. Sci..

[33] Pikal M.J., Shah S. (1990). The collapse temperature in freeze drying: Dependence on measurement methodology and rate of water removal from the glassy phase. Int. J. Pharm..

[34] Greco K., Mujat M., Galbally-Kinney K.L., Hammer D.X., Ferguson R.D., Iftimia N., Mulhall P., Sharma P., Kessler W.J., Pikal M.J. (2013). Accurate Prediction of Collapse Temperature using Optical Coherence Tomography-Based Freeze-Drying Microscopy. J. Pharm. Sci..

[35] Ullrich S., Seyferth S., Lee G. (2015). Measurement of shrinkage and cracking in lyophilized amorphous cakes. Part I: Final-product assessment. J. Pharm. Sci..

[36] Ullrich S., Seyferth S., Lee G. (2015). Measurement of shrinkage and cracking in lyophilized amorphous cakes. Part II: Kinetics. Pharm. Res..

[37] Lam P., Patapoff T.W. (2019). Split-cakes, still delicious. PDA J. Pharm. Sci. Technol..

[38] Esfandiary R., Gattu S.K., Stewart J.M., Patel S.M. (2016). Effect of Freezing on Lyophilization Process Performance and Drug Product Cake Appearance. J. Pharm. Sci..

[39] Ohori R., Akita T., Yamashita C. (2018). Effect of temperature ramp rate during the primary drying process on the properties of amorphous-based lyophilized cake, Part 2: Successful lyophilization by adopting a fast ramp rate during primary drying in protein formulations. Eur. J. Pharm. Biopharm..

[40] Ohori R., Yamashita C. (2017). Effects of temperature ramp rate during the primary drying process on the properties of amorphous-based lyophilized cake, Part 1: Cake characterization, collapse temperature and drying behavior. J. Drug Deliv. Sci. Technol..

[41] Pikal M.J., Roy M.L., Shah S. (1984). Mass and Heat Transfer in Vial Freeze-Drying of Pharmaceuticals: Role of the Vial. J. Pharm. Sci..

[42] Lewis L.M., Johnson R.E., Oldroyd M.E., Ahmed S.S., Joseph L., Saracovan I., Sinha S. (2010). Characterizing the Freeze–Drying Behavior of Model Protein Formulations. AAPS PharmSciTech.

[43] Cleland J.L., Lam X., Kendrick B., Yang J., Yang T., Overcashier D., Brooks D., Hsu C., Carpenter J.F. (2001). A specific molar ratio of stabilizer to protein is required for storage stability of a lyophilized monoclonal antibody. J. Pharm. Sci..

