# Supplementary material for: Be Aggressive! Amorphous Excipients Enabling Single-Step Freeze-Drying of Monoclonal Antibody Formulations
Source: Pharmaceutics. 2019 Nov 17;11(11):616. doi: 10.3390/pharmaceutics11110616 (PMC6920930; doi:10.3390/pharmaceutics11110616)
Supplement: Supplementary file 1 [file pharmaceutics-11-00616-s001.pdf]

pharmaceutics

Supplementary Materials: Be Aggressive! Amorphous Excipients Enabling Single-Step Freeze-Drying of Monoclonal Antibody Formulations

Christina Haeuser, Pierre Goldbach, Joerg Huwyler, Wolfgang Friess and Andrea Allmendinger

Table S1 shows the composition of the different formulations. F_S served as reference formulation throughout this study.

Table S1. Formulation composition. All formulations were prepared in 20 mM histidine/histidine-HCl buffer pH 6.0 containing 0.02% polysorbate 20.

Formulation	mAb (mg/mL)	Sucrose (mg/mL)	HPBCD (mg/mL)	PVP (mg/mL)
F_S	10<br>50	80	-	-
F_{CD}	10<br>50	80	-	-
$F_{CD/P/S}$	10<br>50	24	39.2	16.8
$F_{CD/S}$	10<br>50	24	56	

HPBCD: 2-hydroxypropyl-betacyclodextrin.

PVP: Polyvinylpyrrolidone.

Figure S1 shows the reconstitution times of 10 mg/mL and 50 mg/mL mAb formulations freeze-dried with different cycle parameters. For 10 mg/mL mAb formulations reconstitution times were comparable for all formulations and cycle conditions. Reconstitution of 50 mg/mL mAb formulations took generally longer and reconstitution of F_S took around 80 s (s) longer compared to F_{CD}, $F_{CD/P/S}$, and $F_{CD/S}$, independent of the cycle employed.

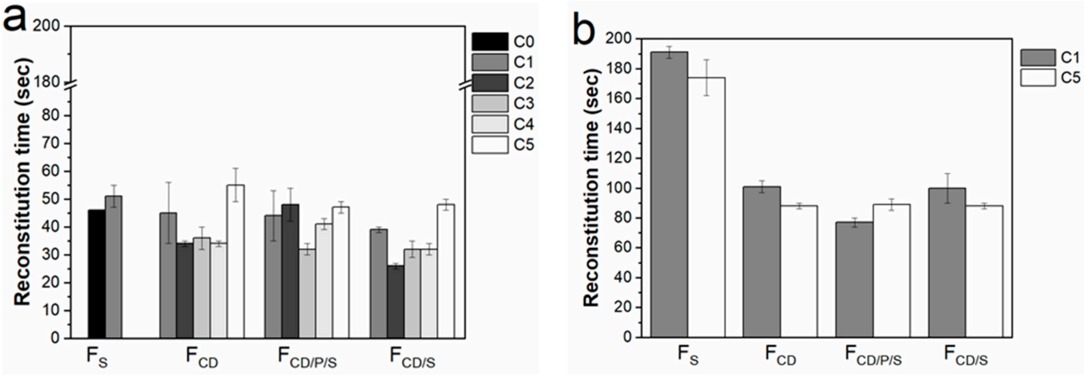

Figure S1. Reconstitution time of lyophilisates. **a**) 10 mg/mL mAb formulations and **b**) 50 mg/mL mAb formulations freeze-dried with different cycles.

Figure S2 shows the specific surface areas of 10 mg/mL and 50 mg/mL mAb formulations freeze-dried with different cycle parameters. In general, F_S showed a slightly lower specific surface area compared to F_{CD}, $F_{CD/P/S}$, and $F_{CD/S}$. In addition, F_S at 10 mg/mL mAb, when freeze-dried with C1 had a lower specfic surface area, which is indicative for the collapsed lyophilisate.

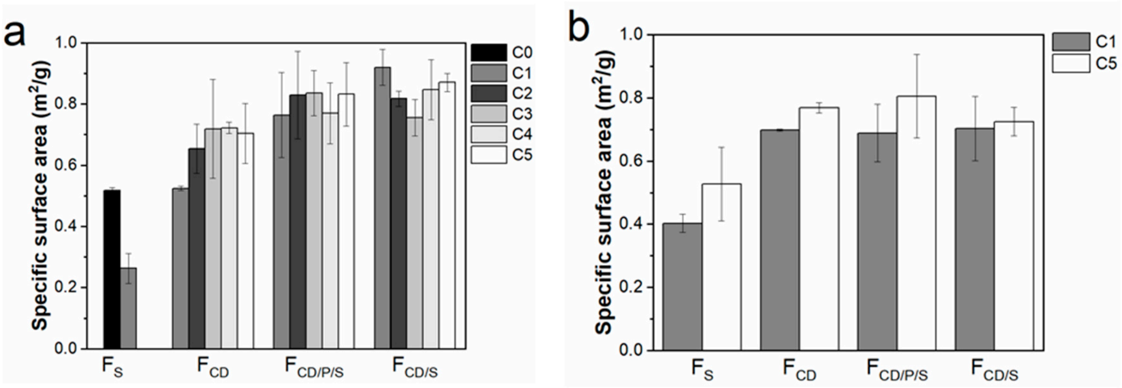

Figure S2. Specific surface area of lyophilisates. **a**) 10 mg/mL mAb formulations and **b**) 50 mg/mL mAb formulations freeze-dried with different cycles.

Figure S3 shows mAb stability after 3 months 40 °C of 10 mg/mL lyophilisates that were spiked to different residual moisture levels to investigate whether very low residual moisture levels might be detrimental to mAb stability during storage. Stability was not affected in a moisture level range between 0.2% and 2%.

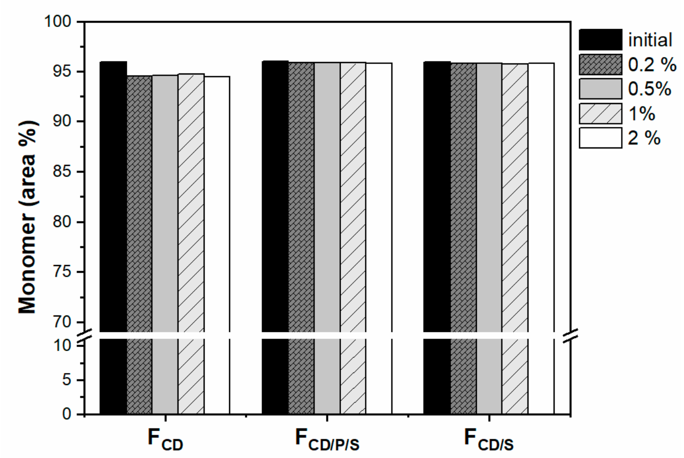

Figure S3. Protein stability dependent on residual moisture (in %). Monomer content by SE-HPLC is given for 10 mg/mL formulations after freeze-drying and after storage for 3 months at 40 °C.

© 2019 by the authors. Submitted for possible open access publication under the terms and conditions of the Creative Commons Attribution (CC BY) license (http://creativecommons.org/licenses/by/4.0/).
